# Impact of Maternal Health Behaviours and Social Conditions on Infant Diet at Age 1-Year: Results from a Prospective Indigenous Birth Cohort in Ontario, Canada

**DOI:** 10.3390/nu14091736

**Published:** 2022-04-22

**Authors:** Gita Wahi, Julie Wilson, Melanie Burning, Stephanie George, Phyllis Hill, Janet Homer, Laurie Jacobs, Ashley Lickers, Sharon Smoke, Albertha D. Davis, Dipika Desai, Susan M. Jack, Natalie Williams, Russell J. de Souza, Sonia S. Anand

**Affiliations:** 1 Department of Pediatrics, McMaster University, Hamilton, ON L8S 4K1, Canada; 2 Department of Health Research Methods, Evidence and Impact, McMaster University, Hamilton, ON L8S 4L8, Canada; jacksm@mcmaster.ca (S.M.J.); desouzrj@mcmaster.ca (R.J.d.S.); anands@mcmaster.ca (S.S.A.); 3 Six Nations Birthing Centre, Six Nations of the Grand River, Ohsweken, ON N0A 1M0, Canada; juliewilson@sixnations.ca (J.W.); hbfhv3@sixnations.ca (M.B.); sgeorge@sixnations.ca (S.G.); phill2@sixnations.ca (P.H.); jhomer@sixnations.ca (J.H.); ljacobs@sixnations.ca (L.J.); mcms5@sixnations.ca (A.L.); ssmoke@sixnations.ca (S.S.); 4 Six Nations Health Services, Six Nations of the Grant River, Ohsweken, ON N0A 1M0, Canada; ddavis@sixnations.ca; 5 Population Health Research Institute, Hamilton, ON L8L 2X2, Canada; dipika.desai@phri.ca; 6 School of Nursing, McMaster University, Hamilton, ON L8S 4L8, Canada; 7 Chanchlani Research Centre, Department of Medicine, McMaster University, Hamilton, ON L8S 4L8, Canada; natcampb@mcmaster.ca

**Keywords:** Indigenous health, diet, social determinants of health, midwifery

## Abstract

Background: Understanding the impact of maternal health behaviours and social conditions on childhood nutrition is important to inform strategies to promote health during childhood. Objective: To describe how maternal health sociodemographic factors (e.g., socioeconomic status, education), health behaviours (e.g., diet), and traditional health care use during pregnancy impact infant diet at age 1-year. Methods: Data were collected from the Indigenous Birth Cohort (ABC) study, a prospective birth cohort formed in partnership with an Indigenous community-based Birthing Centre in southwestern Ontario, Canada. 110 mother-infant dyads are included in the study and were enrolled between 2012 and 2017. Multiple linear regression analyses were performed to understand factors associated with infant diet scores at age 1-year, with a higher score indicating a diet with more healthy foods. Results: The mean age of women enrolled during pregnancy was 27.3 (5.9) years. Eighty percent of mothers had low or moderate social disadvantage, 47.3% completed more than high school education, and 70% were cared for by a midwife during their pregnancy. The pre-pregnancy body mass index (BMI) was <25 in 34.5% of women, 15.5% of mothers smoked during pregnancy, and 14.5% of mothers had gestational diabetes. Being cared for by an Indigenous midwife was associated with a 0.9-point higher infant diet score (*p =* 0.001) at age 1-year, and lower maternal social disadvantage was associated with a 0.17-point higher infant diet quality score (*p =* 0.04). Conclusion: This study highlights the positive impact of health care provision by Indigenous midwives and confirms that higher maternal social advantage has a positive impact on child nutrition.

## 1. Background

The social determinants of health (SDoH) are defined by the World Health Organization as: “conditions in which people are born, grow, live, work, and age” [1]. There is ample evidence to suggest that social risks including low income, lack of housing, poor literacy, inaccessibility to transportation, and food insecurity have a negative impact on health outcomes [2]. These social and economic factors work upstream to influence the health of a population and are important contributors to the increasing global prevalence of non-communicable diseases, including obesity [3,4].

Determinants of health range from proximal factors such as biological factors and individual lifestyle behaviours, to social and community factors and distal determinants such as systems of policy and education [5]. Key proximal determinants of obesity and other non-communicable diseases are health-related behaviours including diet. Among children, dietary practices may impact the trajectory of health outcomes across the life course and contribute to the rising prevalence of childhood obesity [6,7]. However, childhood nutrition is influenced by a complex interplay of factors including but not limited to household income, parental education, and access to services [8,9,10]. Despite widespread acceptance that obesity is a product of proximal, intermediate, and distal factors, the focus of obesity prevention programs over the last 25 years continues to focus on individual-level or proximal factors [5]. Characterizing the impact of the distal SDoH on childhood nutrition and obesity, and identifying protective factors are important to inform strategies to promote healthy nutrition during childhood and prevent childhood obesity [11].

In Canada and other countries where historical context is rooted in the legacy of European colonization, it would be incomplete to discuss the impact of the SDoH on obesity and related factors without considering the context of Indigenous peoples. As outlined by Reading and Wien in the National Collaborating Centre for Aboriginal Health, overarching determinants of health that significantly impact the health of Indigenous peoples in Canada include: colonialism, racism, and social exclusion [12]. In Canada, Indigenous peoples include First Nations, Métis, and Inuit peoples; they are the original inhabitants of Turtle Island, the land that is now called Canada [13]. Colonization and government-led genocide have eroded traditional practices, language, and ways of life among Indigenous communities in Canada and have led to poor health outcomes [14,15]. Examples of these health disparities include rates of infant mortality, which in Canada are higher among Indigenous peoples compared to non-Indigenous Canadians [16]. Indigenous children and adults also have higher rates of chronic diseases including obesity, cardiovascular disease, and diabetes [17,18,19,20]. The resiliency and strength of Indigenous communities are grounded in connections with community, culture, and traditions [21], and these cultural practices are important influences on the dietary patterns of individuals and communities. The root of these health inequities must be put in the context of the government-sponsored socio-political atrocities experienced by Indigenous communities [12].

A recent systematic review of obesity prevention programs highlights the scarcity of effective programs for Indigenous children and noted a modest number of studies that showed beneficial dietary outcomes [22]. However, a dimension that is often not addressed is cultural continuity, or being connected to one’s culture, which has shown to be protective against chronic diseases, such as type 2 diabetes, among First Nations communities [21]. Further understanding of contextual factors that serve as barriers to or facilitators of optimal early life nutrition may help inform the design of future programs promoting healthy nutrition and preventing obesity. We sought to understand the factors that influence the diet of young children enrolled in an Indigenous birth cohort study (ABC) [23], with the understanding that nutrition is an important contributor to child health and the SDoH can impact dietary practices [24,25,26,27]. Specifically, in this analysis, we aim to describe the health behaviours of mothers during pregnancy and infants at age 1-year, and understand how contextual factors including social disadvantage, maternal education, diet, and access to traditional health care practices are associated with infant diet, at age 1-year.

## 2. Methods

Study design: This is an analysis of a prospective birth cohort study. Pregnant women and their newborns living in or nearby a First Nations community in southern Ontario, Canada were enrolled in the ABC study and followed prospectively to the age of 3 years between 2012 to 2017 [23]. This is one of the first birth cohorts specifically for mothers and infants of Indigenous ancestry in Canada and gives a comprehensive understanding of factors in early life that may impact the health and wellbeing of Indigenous children of the community [23]. The cohort included women who self-identified as Indigenous ancestry in their 2nd trimester of pregnancy. Women who conceived using artificial methods including in-vitro fertilization or ovarian hyperstimulation, women carrying more than one fetus, surrogate mothers, or women with severe chronic medical conditions including active cancer, and severe infectious diseases, were excluded.

Setting: The setting includes the Six Nations of the Grand River in Brant County, Ontario, Brantford General Hospital, Brantford Ontario, Hamilton Health Sciences Corporation, Hamilton, Ontario, and Tsi Non:we Ionnakeratstha (a Mohawk language word translating to: “The Place They Will Be Born”) Ona:grahsta’ (a Cayuga Language Word translating to: “A Birthing Place”)—the Six Nations Birthing Centre. The Six Nations Birthing Centre was established in 1996 on the Six Nations of the Grand River Territory and is a place where Indigenous midwives provide traditional midwifery care and services.

Research-community collaboration: A community-based participatory research framework guided this research to redistribute power held within non-Indigenous research methods [28,29]. Academic researchers and community members identified areas of focus for the ABC study and strategies for addressing areas of concern, specifically childhood obesity. This specific study question was co-created with community Indigenous midwives and academic researchers in the fall of 2020. To accommodate social distancing due to COVID-19, information was exchanged over email, web-based surveys, and teleconferences. Initially, midwives completed online surveys that identified their areas of interest including time periods, e.g., pregnancy, birth, early childhood, as well as topics that were important to them. Subsequently, two group meetings were held over teleconference to discuss survey results and refine the questions. Through discussion of priorities, available cohort data, and previous projects completed together, a list of topics and questions of interest were compiled. Generated within the list of ideas was a priority of understanding the impact of SDoH on child outcomes, including nutrition, and the impact of the care of the traditional Indigenous midwifery practices. Follow-up discussion of the data framed analysis and the interpretation of results.

Data collection: The women enrolled in the ABC study underwent a baseline assessment in their second trimester of pregnancy with subsequent data collection for mothers and infants at birth, 6 weeks, 6 months, then annually until age 3 years. Data collection methods and measures are previously published [23]. Data collected for mothers and offspring included demographics, medical and social history, as well as lifestyle behaviours including nutrition, physical activity, and sleep. For the purposes of this study, data is included from the maternal baseline visit during the second trimester of pregnancy and follow-up visit at 1-year. Healthy lifestyle characteristics including physical activity and screen time behaviours were self (or parent)-reported, from the mother’s baseline visit during pregnancy and from the child’s 1-year follow-up visit. Infant sleep duration for a 24-h period was collected from a parent-reported survey at age 1-year. Dietary data were collected with previously validated semi-quantitative food frequency questionnaires (FFQ) or when not available a 19-item short qualitative food frequency questionnaire [30,31]. A previous validation study of a similar approach in adults found a good correlation between the short FFQ and the comprehensive FFQ for seafood, dairy, egg, fruits, potatoes, grains, soft drinks, and processed meat (Spearman rank correlation > 0.5); a moderate correlation for meat, sweets, vegetables, protein, and carbohydrates (Spearman rank correlation: 0.3–0.5); and a weaker correlation for total fat measured by SFFQ and CFFQ (Spearman rank correlation < 0.3) [32]. The maternal FFQs were administered to the mother at the baseline visit during pregnancy and at 1 year follow-up. The infant FFQ collected at the age of 12 months was semi-quantitative and built from existing instruments including the Avon Longitudinal Study of Parents and Children (ALSPAC) [33] and the Study of Health Assessment and Risk Evaluation in Aboriginal People (SHARE-AP) [20]. The FFQ asks the respondent to recall, on average, how many times a specified food (or group of similar food items) was consumed over a specified time (e.g., once or more per day; once per week; once or less per year). Individual foods were classified into food groupings to account for similar types of foods, for example, the grouping ‘fish and seafood’ included items on the FFQ such as ‘fish in batter’, ‘other white fish’, ‘oily fish’, and ‘canned fish’. We then calculated the average number of times the food was consumed per day. If the consumption was reported as a “per week” time-unit, by the participant the amount was divided by 7 (e.g., once per week = 0.14 times per day), if reported as “per month”, divided by 30.44 (e.g., once per month = 0.03 times per day) and if reported “per year”, divided by 365.25 (e.g., once per year = 0.003 times per day).

Through discussions with community members from the study team and informed by prior focus groups and interviews [25], we created two lists of foods. The first was “healthy foods” (HF), these were foods that were encouraged in the diet of a young child, and the second group was “less healthy foods” (LHF) that were advised to consume infrequently. The HF category included the following food groups: vegetables, fruit, meats, fish, breastmilk, traditional foods, whole grains, and nuts. The LHF category included the following food groups: processed foods (e.g., pizza, hot dogs, deli meat), sugar-sweetened beverages (SSBs), sweets, and refined grains.

The distribution of consumption frequencies for each of the healthy and less-healthy foods was placed into quartiles to generate healthy and less healthy diet scores. Consuming more than 75% of a specific healthy food (i.e., >75th percentile) was equal to a score of 4 for a food; between 50–75%, a score of 3; and 25 to <50%, a score of 2, and <25th percentile, a score of 1. Breastfeeding was assigned by duration in months. The highest possible HF score was 32 (8 foods × 4), and the lowest was 8 (8 foods × 1). The same procedure was used for the LHF score, for which the highest possible score was 16 (4 foods × 4), and the lowest was 4 (4 foods × 1). Next, the HF and LHF scores were divided into quartiles, named the qHF and qLHF scores, which accounted for the different number of foods in each score. We used a similar system of scoring as we did for the individual scores, where the lowest quartile was assigned 1 and then the highest quartile was assigned 4. Infant diet score (iDS) was defined as the difference between qHF and qLHF. It had a maximum value of 3 and a minimum value of −3, with a higher score signifying a larger number of healthy foods in the diet. The iDS were dichotomized with a value ≥ 0 as a ‘positive diet score’ with more healthy foods and <0 as a ‘negative diet score’ or more unhealthy foods for comparison of maternal and child characteristics between groups.

Contextual factors: We sought to understand the impact of contextual factors on infant diet and the following constructs were chosen to be included in the model based on a review of the literature and guidance from the local midwives: maternal social disadvantage, maternal education, maternal dietary score, and health care provider. Maternal social disadvantage was defined by the social disadvantage index (SDI) [34]. The SDI is a score that combines income, employment, and marital status, developed, and validated with a multiethnic cohort of adults [34]. We calculated the SDI for the mother with a score out of 5 where 0–1 is considered low, 2–3 is considered moderate, and 4–5 as high social disadvantage [34]. Maternal education was self-reported on the baseline questionnaire and dichotomized as less than or equal to high school or more than high school. Maternal diet score was calculated as previously described [30,31]. The health care provider was recorded for each participant, which included an Indigenous midwife at the Six Nations Birthing Centre or another provider, for example, an obstetrician practicing in a western medicine model.

Statistical Analysis: To understand differences in the distributions of contextual factors between infants with positive and negative diet scores, we performed Fisher’s exact test to compare the distributions of categorical variables, and *t*-tests or Mann-Whitney U tests to compare means of continuous variables. We performed multiple linear regression analyses to identify factors associated with infant diet scores. Unadjusted and adjusted linear regression analyses were presented with beta coefficients, standard errors, and *p* values. First, each potential predictor variable was tested for its univariate association with infant diet score. Next, those variables significantly associated with diet score (simple regression *p* < 0.10) were included in the multiple regression model. The variance inflation factor was used to understand the possibility of multicollinearity among all the variables included in the models (<5 indicated insignificant collinearity). The α was set at <0.05 for all statistical tests, with no adjustment for multiplicity. All analyses were performed using IBM SPSS Statistics for Macintosh, Version 26.0. IBM Corporation (Armonk, NY, USA).

## 3. Results

There were 157 women recruited into the cohort between 2012 and 2017, and two participants withdrew, leaving 155 mother-infant dyads. Baseline maternal and infant characteristics are shown in Table 1. Of the dyads followed through infant age 1-year, 71% (*n =* 110) had complete food-frequency data for mothers at baseline and infants at age 1-year. The average age of mothers in the cohort was 27.3 (SD = 5.9) years. A total of 14.5% of mothers had gestational diabetes during second-trimester screening using glucose tolerance tests and 34.5% of mothers had a pre-pregnancy body mass index (BMI) of less than 25. Overall, 15.5% of mothers smoked during pregnancy. Self-reported minutes of exercise/day was 35.8 (52.6), and they reported 196.0 (147.0) minutes/day of screen time. The average maternal diet index was 1.3 (7) and 78.7% of mothers consumed vitamin supplements during pregnancy. With regards to contextual factors, 80% of mothers had a low or moderate social disadvantage with a mean (SD) SDI score of 2.13 (1.52) and 47.3% had completed more than high school education. Most participants (70%, *n =* 77) received prenatal care at the Birthing Centre, and the others with care providers including local obstetricians or primary health care providers. Of the infants, 47.3% were female, mean gestational age was 38.0 (1.7) weeks, mean birth weight was 3578 (486.9) grams and 71.8% were birthed by vaginal deliveries.

One year assessment: At the 1-year visit, 60.9% of infants had been breastfed for >6 months, and the mean infant diet score was −0.04 (1.2), with 29% (*n* = 32) having a diet score greater than 0 which we classified as a ‘positive diet score’. The duration of mother-reported infant physical activity was 222.1 (179.7) minutes per day and exposure to screens during the day was 5.0 (5.0) minutes per day. Sleep, for children at age 1-year in a 24-h period, was reported as 750.4 (102.4) minutes (~12.5 h).

Table 2 compares contextual factors between infants with positive (*n* = 32; mean = 1.4, SD = 0.6) and negative (*n* =78; mean = −0.6, SD = 0.9) iDS. The proportion of mothers who had completed more than a high school education was higher in the positive infant diet score group compared to the negative infant diet score group (69% vs. 39%, *p =* 0.014). There were more mothers experiencing low or moderate SDI (vs. high) in the positive infant diet score compared to the negative infant diet score group (92% vs. 68%, *p =* 0.016). A significantly higher proportion of women had a household income > $60,000 annually in the positive iDS than in the negative iDS group (41.9% vs. 22.7%, *p =* 0.047). More mothers in the positive iDS group were cared for at the Birthing Centre than in the negative iDS group (87.5% vs. 62.3%, *p =* 0.012). The maternal diet score was higher among infants with a positive versus negative diet score (*p =* 0.01). There were no other significant differences in the groups with the positive and negative infant diet scores for other maternal characteristics. There were no differences in infant characteristics between high and low iDS groups, except for the gestational age being slightly lower in the positive iDS group (38.4 vs. 39.1 weeks; *p =* 0.05).

Results of the univariate and multiple linear regression analyses are shown in Table 3. In the multiple linear regression model, care provided by Indigenous midwives at the Birthing centre was associated with higher infant diet scores (B = 0.9, SE 0.25; *p =* 0.001). Additionally, a 1-unit lower SDI score, (i.e., low social disadvantage) was associated with higher iDS (B = 0.17 (0.08) *p =* 0.039). Maternal diet was significant in the univariate analysis but did not remain significant in the multiple linear regression analysis. Maternal education was not significantly associated with infant diet score.

## 4. Discussion

In this paper, we describe the demographic, health, and health behaviour characteristics of a cohort of Indigenous mothers and infants in Ontario, Canada [23]. We show a positive influence of low social disadvantage and pregnancy-related health care provision by Indigenous midwives on infant diet at one year after birth, reflected in a higher infant diet quality score.

Most participants in this study received care through the Birthing Centre. The availability of holistic and community services spanning pregnancy to early childhood may be a mechanism for higher dietary scores among those cared for at the Birthing Centre. For example, within the Birthing Centre, co-existing services and guidance from an Elders “Grandparents group” provide support for mothers and families from pregnancy into early childhood with services, including: prenatal classes, Healthy Babies/Healthy Children services led by community health workers, breastfeeding support from an Indigenous lactation consultant, and ‘Mom and Tot’ groups. This highlights that Indigenous midwifery care is an important and central part of health services, not only for the health care services they provide, but also for the culturally-appropriate support services they facilitate [35]. Access to perinatal health care is essential for all mothers, and for care to be effective, it must be provided in a manner consistent with cultural values and beliefs [36]. In Canada, disparities in access to prenatal and birthing care for Indigenous communities have led to poor health outcomes for Indigenous mothers and infants [37,38]. This study provides a strong argument for ongoing funding, development, and implementation of localized, culturally-sensitive antenatal and early childhood care within Indigenous communities and with access for all Indigenous mothers.

We found that lower maternal social disadvantage during pregnancy was associated with a higher quality infant diet at age 1-year. Additionally of note, among infants with a positive diet score, it was more likely that mothers had higher income, more education, and higher diet score. In this study, the social disadvantage index was used as a measure of socio-economic status. It has been observed that adults with a higher SDI score have more cardiovascular risk factors [34]. The SDI was developed over 20 years ago, as a measure of socioeconomic disadvantage. Anand et al. recently showed its utility in a multi-community evaluation of cardiovascular risk factors in eight First Nations communities across Canada [39]. We believe this measure is still valid as the circumstances of participants in our cohort are comparable to previous cohorts, as demonstrated with similar SDI scores [34,39]. A plausible explanation linking social disadvantage to infant diet is the hypothesis that it is a proxy for household food insecurity, or inadequate access to food. Though we did not directly measure household food insecurity in our study, it may be part of the pathway linking SDI to infant dietary score and has previously been shown to be related to poor general health in children [40,41]. Further, the prevalence of food insecurity among Indigenous communities in Canada is high [42] and has a significant impact on health outcomes [43]. This study is adds to the argument to consider the social determinants of health for families with young children as a key pathway to improved child health outcomes.

We considered whether the participants in our sample had health profiles comparable to those of other populations. Compared with a retrospective chart review of 453 mothers and infants from the Six Nations Birthing Centre between 2005–2010, the ABC study included mothers of older age, lower pre-pregnancy BMI, and with a lower rate of smoking during pregnancy, but higher prevalence of gestational diabetes mellitus (14.5% vs. 4.7%) [44]. Considerations for the difference in rates of GDM include that the retrospective chart review only included mothers cared for at the Birthing Centre, whereas the ABC study also included mothers cared for by obstetricians, and therefore may have had different medical profiles; further in the ABC analysis OGTTs were administered in a standardized manner whereas the method of testing was not collected from the retrospective study. In a systematic review, Voaklander et al., describe higher rates of diabetes during pregnancy among Indigenous mothers in Canada, the United States, Australia, and New Zealand, although interestingly the incidence of GDM is lower when compared to a recent South Asian study reported in Canada [45].

Infants in our cohort had a lower birth weight and gestational age compared to observations from the Birthing Centre chart review [44]. Over 60% of infants were breastfed for more than 6 months, which is considerably higher than the ~33% of First Nations mothers living off-reserve as reported by Statistics Canada’s 2006 Aboriginal Children’s Survey [46].

Diet is a key determinant in the trajectory of non-communicable chronic diseases including obesity [47,48]. For example, children with a higher intake of processed foods and sugar-sweetened beverages are at greater risk of developing obesity. The diet score created in this study was based on direct input from community members and consistent with a healthful traditional dietary pattern for young children [25]. Despite the rich traditions, heterogeneity, and diversity between Indigenous communities, similar themes emerge from distinct communities regarding traditional food consumption among children [49]. Specifically, traditional foods are an important source of nutrients and important for health, but not commonly consumed. A study with Inuit children attending daycare in Nunavut showed that only 3% of 245 children consumed traditional foods, but those that did had a higher intake of macro- and micronutrients [50]. Other studies have corroborated this including a study with Indigenous infants from rural Australia, where a cross-sectional study using 24-h dietary recalls found that >50% of children consumed some traditional foods, and these foods were more nutrient-dense [51]. However, they also observed that traditional food consumption decreased with times of food insecurity [51]. A systematic review of diets of school-age Indigenous youth in Canada summarized 24 articles and found 7 that reported on traditional food consumption [52]. In these 7 studies, traditional foods were an important dietary source of micronutrients for children, but intake was infrequent in most settings [52]. Further, traditional food consumption has been associated with positive health outcomes as demonstrated by the Six Nations of the Grand River community-led, Haudenosaunee traditional food initiative, *Entsisewata’karí:teke* (“You Will Be Healthy Again”) [53]. Access to traditional foods should therefore be considered in future programs and evaluations of programs concerning infant diet in Indigenous communities.

Strengths of this study include leveraging the prospective cohort study design to understand relationships between family context and child health outcomes. The other key component of this study was the co-creation of the study question by Indigenous midwives, community members, and the academic study team. The development of the study question allowed for the exploration of the impact of traditional midwifery care, an important consideration for community study team members. The primary outcome of infant diet was directly informed by qualitative interviews and a community focus group [25] and further embeds the results within the context of the community, which will improve the usefulness of the study findings in practice. Limitations to this study include the attrition of the cohort at the 1-year visit (71% follow-up at 1-year). Further, a limitation of this work is that the dietary outcome is based on a self-reported food frequency questionnaire [54]. Sources of measurement error in such instruments have been well-documented. There are also limits to the generalizability of the study results due to the non-randomly selected convenience sample, as well as the fact that only one community was included in the cohort. Therefore, the study results may not be applicable to other communities given the rich, diverse, traditions, and ways of life unique to Indigenous communities in Canada.

In conclusion, this study demonstrates the impact of important protective factors of higher social advantage and access to a model of healthcare that is congruent with traditional ways of life. The academic-community partnerships among the study team led to important findings relevant to care providers.

## Figures and Tables

**Table 1:** Maternal characteristics at baseline and infants at age 1-year.

Characteristic	Total (*n =* 110)
Maternal	
Age at baseline ^a^	27.3 (5.9)
Self-report exercise (min/day) ^b^	30 (50)
Self-reported screen time (min/day) ^b^	180 (215)
Diet index score ^b^	1.3 (7)
Gestational diabetes during this pregnancy ^c%^	14.5
Pre-pregnancy Body mass index (BMI) ^a^	27.6 (6.9)
Pre-pregnancy BMI <25 ^c%^	34.5
Social disadvantage index (low/moderate) ^c^	80
SDI ^a^	2.13 (1.52)
Maternal education more than high school ^c%^	47.3
Maternal smoking ^c%^	15.5
Maternal prenatal vitamin use ^c%^	78.7
Birthing centre ^c%^	70
Infant	*n* = 110
Gestational age (weeks) ^a^	38.9 (1.7)
Sex (female) ^c%^	47.3
Birth weight (grams) ^a^	3578 (486.9)
Birth mode (vaginal) ^c%^	71.8
Age 1-year visit (months) ^a^	1.3 (0.4)
Weight at 1-year (kg) ^a^	11.3 (2.6)
Breastfeeding (>6 months) ^c%^	60.9
Self-reported Physical activity (min/day) ^b^	150 (235)
Screen (TV) on at home during day (min/day) ^b^	5.0 (5.0)
Sleep (min/24 h) ^a^	750.4 (102.4)
Diet score ^a^	−0.04 (1.2)

^a^ mean (standard deviation); ^b^ median and interquartile range; ^c^ count (%).

**Table 2:** Maternal and infant characteristics at age 1-year in positive and negative diet score.

Characteristic	Infant Positive Diet Score (*n* = 32)	Infant Negative Diet Score (*n =* 78)	*p*
Maternal			
Age at baseline ^a^	28.4 (5.1)	26.8 (6.2)	0.21
Maternal smoking ^b^	12.5%	16.6%	0.77
Gestational Diabetes Mellitus ^b^	18.8%	12.8%	0.55
SDI * (low/moderate) ^b^	92.3%	67.8%	**0.016**
-SDI (married) ^b^	75%	64.9%	0.37
-SDI (employed) ^b^	65%	50%	0.09
-SDI (income > 60,000) ^b^	41.9%	22.7%	**0.047**
Maternal education more than high school ^b^	69%	38.5%	**0.014**
Maternal pre-pregnancy BMI <25 ^b^	35.7%	36.8%	1.00
Maternal Diet score ^c^	2.7 (6)	0.5 (6)	**0.01**
Birthing centre ^b^	87.5%	62.3%	**0.012**
Infant			
Gestational age ^a^	38.4 (2.3)	39.1 (1.2)	0.05
Sex (female) ^b^	56.2%	41.7%	0.18
Birth weight (grams) ^a^	3520.9 (523.9)	3604.4 (471.3)	0.44
Birth mode (vaginal) ^b^	87%	89.6%	0.73
Age 1-year visit (years) ^a^	1.19 (0.21)	1.28 (0.41)	0.25
Weight at 1-year (kg) ^a^	11.2 (2.0)	11.3 (2.8)	0.84
Breastfeeding >6 months ^b^	86.7%	56.1%	**0.003**
Infant Diet score ^a^	1.38 (0.55)	−0.62 (0.87)	**<0.001**

* The Social Disadvantage Index (SDI) was developed from an ethnically diverse sample of Canadian adults and takes into account income, marital and employment status [32]. A score out of 5 is calculated. A score of 0–1 is considered low 2–3 considered moderate and >4 as high social disadvantage [32]. ^a^ mean (standard deviation); ^b^ count (%) ^c^ median (interquartile range).

**Table 3:** Multiple linear regression models for infant diet score.

Factor	Univariate Analysis Beta (Standard Error) *p*	Multivariate Analysis Beta (Standard Error) *p*
Maternal SDI	−0.21 (0.08) *p =* 0.007	−0.17 (0.08) ***p =* 0.039**
Maternal education	0.18 (0.14) *p =* 0.28	-
Maternal diet score	0.04 (0.02) *p =* 0.004	0.03 (0.02) *p =* 0.11
Care provider	0.94 (0.24) *p* ≤ 0.001	0.90 (0.25) ***p =* 0.001**

## Data Availability

Restrictions apply to the availability of these data. Any requests for de-identified data presented in this study will be handled on a case-by-case basis, by the corresponding author in consultation with the senior author and partners from Six Nations of the Grand River, consistent with OCAP^®^ principles.
